# Continuous Extradural Infusion of Ropivacaine in a Cat with Severe Abdominal Trauma

**DOI:** 10.3390/ani15162378

**Published:** 2025-08-13

**Authors:** Dany Elzahaby, Isabelle Iff

**Affiliations:** 1 Vetsuisse Faculty, Department of Clinical Veterinary Medicine, Section of Anaesthesiology and Pain Therapy, University of Bern, 3012 Bern, Switzerland; isabelle.iff@unibe.ch; 2 Vet Doc Iff GmbH, 4600 Olten, Switzerland

**Keywords:** feline pain, ropivacaine, toxicity, catheter, infusion, CRI, cat, epidural

## Abstract

Continuous extradural infusions (CEIs) provide sustained analgesia, avoiding fluctuations seen with bolus administrations. An extradural catheter was placed in a cat with severe abdominal trauma following a vehicular accident. To provide sustained pain relief, a CEI of 0.5% ropivacaine at 0.18 mg/kg/h or 0.036 mL/kg/h was initiated, resulting in improved analgesia, as shown by lower scores on both the Feline Grimace Scale (FGS) and the Glasgow Composite Measure Pain Scale-Feline (CMPS-Feline). During treatment, the cat developed hypersalivation, which persisted after catheter removal at 72 h. Eventually, the cat’s condition deteriorated, with signs of sepsis leading to euthanasia due to a poor prognosis. Potential contributors to the cat’s decline included systemic infection, local anaesthetic systemic toxicity, or opioid-related effects. This report describes the placement of an extradural catheter in a cat, highlighting the potential of CEI in feline pain management while emphasising the need for further research on its pharmacokinetics and safety.

## Simple Summary

This case report describes the use of a continuous extradural infusion (CEI) of 0.5% ropivacaine to manage pain in a cat that suffered severe abdominal trauma after being hit by a vehicle. An extradural catheter was placed to deliver ropivacaine at a constant rate, which led to improved pain control as measured by validated feline pain scales. While initial bolus injections of ropivacaine provided only short-term relief, the CEI maintained more consistent analgesia. However, the cat later developed hypersalivation and clinical deterioration, eventually diagnosed as likely sepsis. Although local anaesthetic toxicity was considered, it was deemed less likely. This report describes the analgesic potential of a CEI in feline pain management while calling for further studies on its safety and pharmacokinetics.

## 1. Introduction

In veterinary medicine, extradural anaesthesia is used to desensitise the hindlimbs, abdomen, and thorax, effectively alleviating pain in animals following traumatic injury to these regions [1,2,3]. This standard technique involves extradural administration of short- or long-acting local anaesthetics, often in combination with adjunctive agents such as opioids or α2-adrenergic agonists [1,2,3]. While local anaesthetics are typically administered as a bolus, they can alternatively be administered as a continuous extradural infusion (CEI) following placement of an extradural catheter.

In human medicine, the use of CEIs with drugs like ropivacaine and bupivacaine has been extensively documented [4,5,6]. They offer the benefits of sustained analgesia and improved patient comfort by circumventing the sensory fluctuations associated with intermittent bolus dosing [5]. Despite robust evidence in human practice, the veterinary literature is very limited [7].

Ropivacaine, a long-acting amino amide local anaesthetic, has gained popularity in veterinary anaesthesia [2]. Although similar in clinical profile to bupivacaine, it is less cardiotoxic, less likely to cause CNS toxicity, and less likely to cause motor dysfunction [1,4]. Hence, it has become a common choice for extradural anaesthesia, particularly in cats, which are generally considered to have a narrower safety margin for local anaesthetic drugs [8,9,10]. This report describes the use of a ropivacaine CEI in a cat that sustained severe abdominal trauma and required surgical intervention. To the authors’ best knowledge, this is the first described use of a ropivacaine CEI in a cat.

## 2. Case Description

A 3-year-old female neutered domestic cat, weighing 8.2 kg, with a body condition score (BCS) of 9/9, was admitted to a veterinary teaching hospital following suspected vehicular trauma. The cat was diagnosed with abdominal herniation, necessitating surgical intervention. The cat was bright, alert, and responsive, with vital parameters within normal limits despite evident hindquarter trauma. Preanaesthetic haematology revealed mild leucocytosis with a left-shift neutrophilia, while biochemistry indicated stress-induced hyperglycaemia and moderately elevated alanine transaminase (ALT) levels. The patient was classified as American Society of Anaesthesiologists (ASA) physical status III.

The cat was premedicated intravenously with methadone 0.1 mg/kg (Methadon 10 mg/mL, Streuli Pharma AG, Uznach, Switzerland) and medetomidine 4 µg/kg (Domitor 1 mg/mL, Covetrus AG, Lyssach, Switzerland), followed by preoxygenation via a face mask. Anaesthesia was induced intravenously with propofol 0.5 mg/kg (Propofol MCT 10 mg/mL, Fresenius Kabi, Kriens, Switzerland), followed by ketamine 1 mg/kg (Ketanarkon 100 mg/mL, Streuli Pharma AG, Switzerland) administered slowly over 30 s. Additional propofol was then titrated to effect (total cumulative dose 1.2 mg/kg). The trachea was intubated, and anaesthesia was maintained with isoflurane, adjusted according to clinical requirements (EtIso 0.8% to 1.3%). A 22G Tuohy needle was used to puncture the lumbosacral extradural space, identified via the lack-of-resistance technique with a combination of saline and air. Extradural administration of morphine 0.1 mg/kg (Morphine HCL 10 mg/mL, Sintetica, Mendrisio, Switzerland) and ropivacaine 0.91 mg/kg (Ropivacain 0.5%, 5 mg/mL, Sintetica, Switzerland) with a total volume of 1.6 mL (0.2 mL/kg) followed.

An exploratory laparotomy revealed an extensive rupture of the abdominal wall extending to the last rib, along with significant haematoma formation and a splenic laceration. A splenectomy was performed, and the abdominal hernia was repaired. No additional analgesia was administered, as the monitored parameters (HR, RR, NIBP, SpO_2_, EtCO_2_) remained within acceptable clinical limits throughout the surgery. At the conclusion of the procedure, the patient received meloxicam (Inflacam 5 mg/mL, Virbac, Opfikon, Switzerland) 0.1 mg/kg intravenously.

**Figure 1:**
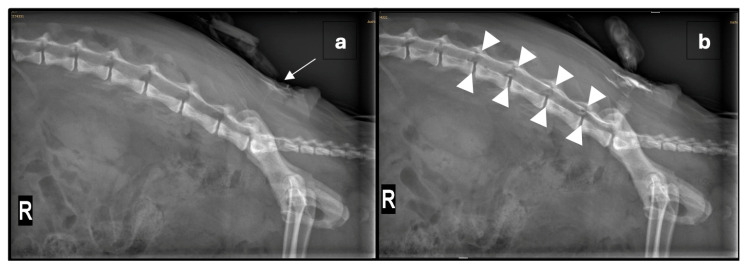
(**a**) Right lateral radiograph of the vertebral column and pelvis showing the positioning of the extradural catheter (arrow) after the injection of a small amount of radio-opaque dye. (**b**) Repeated right lateral radiograph after the administration of 0.5 mL non-ionic iodinated contrast media, diluted in 1 mL NaCl 0.9% solution, through the extradural catheter. The tip of the extradural catheter is not clearly identified; however, by comparing the pre- and post-contrast images, a mild amount of radiopaque material is seen reaching the L3-4 neuroforamina (outlined by arrowheads). Within the abdomen, there is diffuse heterogeneous loss of serosal detail and multifocal areas of free gas following exploratory laparotomy.

Given the severity of the trauma, an extradural catheter (Perifix^®^ One Paed, 24G, 720 mm, B. Braun Medical AG, Melsungen, Germany) was placed using a Tuohy extradural needle (Perican^®^ 20G, B. Braun Medical AG, Germany) with the patient positioned in sternal recumbency. After aseptic preparation of the lumbosacral region and placement of a sterile drape, the needle was inserted at the lumbosacral junction. The extradural space was identified using the lack-of-resistance technique with saline and air, and an aspiration test was negative. A lateral radiograph was then obtained, and the catheter was advanced 5 cm. Diluted non-ionic iodinated contrast medium (0.5 mL iohexolum (Accupaque, 300 mg/mL, GE Healthcare AG, Opfikon, Switzerland) diluted in 1 mL NaCl 0.9%) was injected through the catheter, initially with a low volume to visualise the catheter’s position (Figure 1a). A repeat radiograph, taken after the entire contrast volume was injected, confirmed extradural placement and appropriate contrast spread (Figure 1b). The catheter was then trimmed to 5 cm from its exit site, and a connector as well as a bacterial filter (Perifix^®^ EF 0.2 µm, B. Braun Medical AG, Germany) were attached. The catheter was primed with 0.5% ropivacaine, and the bacterial filter was secured with sutures cranial to the insertion site. These were then covered with a transparent occlusive bandage (Cosmopor transparent, 9 × 7 cm, Hartmann AG, Heidenheim, Germany) and a purpose-made wound protection body used to cover the surgical wounds, as well as the catheter. Subsequently, 1 mL of 0.5% ropivacaine (0.13 mL/kg) was administered, with repeat injections given at 6 h intervals overnight.

**Figure 2:**
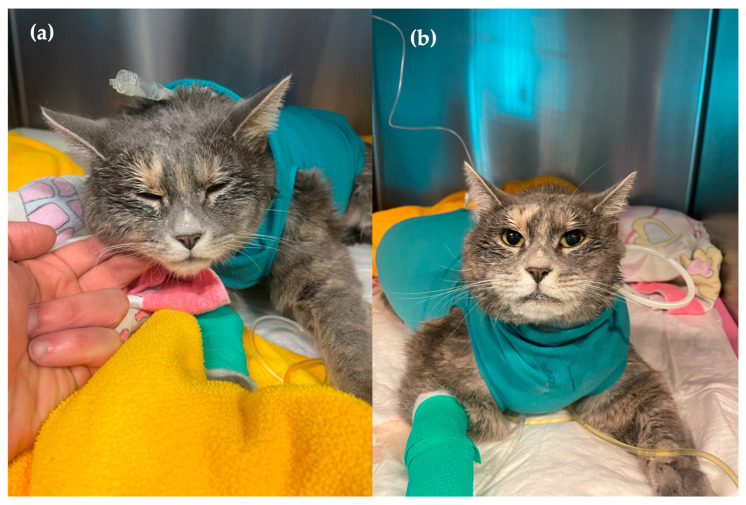
(**a**) A photo of the cat before initiation of a continuous extradural infusion (CEI) of ropivacaine 0.5% at 0.18 mg/kg/h. Note the lowered head position, ear flattening, and squinted eyes. (**b**) A photo of the cat two hours following CEI initiation. Hypersalivation can be observed in this image.

Pain was assessed 14 h post-surgery using the Feline Grimace Scale (FGS) [11] mobile application and the Glasgow Composite Measure Pain Scale-Feline (CMPS-Feline) [12], with scores of 6/10 and 12/20, respectively. Figure 2a shows the cat’s physical appearance. The cat was administered ropivacaine 0.5% 1 mL (0.6 mg/kg/0.13 mL/kg), morphine 0.1 mg/kg, and lidocaine 1.2 mg/kg (Lidocain 2%, 20 mg/mL, Streuli Pharma AG, Switzerland) extradurally. Lidocaine was added to verify the extradural position by assessing anaesthesia shortly after injection. A few minutes after administration, the previously present hindlimb patellar and withdrawal reflexes were absent, thereby supporting correct catheter placement. Clinical signs of pain relapsed approximately three hours post-injection. Repeated extradural administration of ropivacaine 0.5% 1 mL (0.6 mg/kg/0.13 mL/kg) at six-hour intervals resulted in approximately 3 h of efficacy as assessed by the previously mentioned pain scores. Methadone 0.1 mg/kg was administered intravenously as rescue analgesia when clinical signs of pain recurred. During this period, the patient exhibited marked ptyalism, suggestive of nausea. As a result, the patient received intravenous ondansetron 0.3 mg/kg (Ondansetron 2 mg/mL, Labatec Pharma SA, Geneva, Switzerland), maropitant 1 mg/kg (Vetemex 10 mg/mL, Virbac, Glattbrugg, Switzerland), and metoclopramide 0.33 mg/kg (Paspertin 5 mg/mL, Mylan Pharma GmbH, Steinhausen, Switzerland) sequentially.

A CEI of 0.5% ropivacaine was initiated at a rate of 0.18 mg/kg/h (0.036 mL/kg/h) using a syringe pump (Perfusor^®^ Space, B. Braun Medical AG, Germany), which was positioned outside the patient’s cage. The pump was connected to the extradural catheter via an extension set. This set was secured to the top of the cage, allowing the cat to move freely without risking accidental catheter removal. Methadone injections were discontinued, and pain assessment was repeated two hours after starting the ropivacaine infusion to allow adequate time for its effect. The FGS and CMPS-Feline scores were 3/10 and 5/20, respectively. Figure 2b shows the cat’s physical appearance following CEI initiation, with persistent ptyalism still evident at this stage. The withdrawal reflex was absent in the right hindlimb but present in the left. The pain scores remained consistently low until the extradural catheter was removed. In addition to regular pain assessments, the catheter insertion point was also evaluated daily for evidence of inflammation. Blood tests were periodically repeated to evaluate haematocrit, total protein, blood glucose, renal parameters, and other relevant parameters. Visual assessment of the cleanliness of the bandages and body was performed several times a day, alongside monitoring of vital parameters.

After 72 h, pain scores remained low and vital parameters were within normal limits, with heart rate and blood pressure consistently stable. However, ptyalism persisted, and blood values revealed moderate anaemia (Hct: 21%) and hyperglycaemia (15.9 mmol/L). The extradural catheter was removed under aseptic technique using gentle traction and was retrieved intact without complication. Systemic analgesia was continued with intravenous administration of meloxicam 0.05 mg/kg and methadone 0.2 mg/kg. Twenty-four hours following removal of the extradural catheter, the patient’s general condition deteriorated, developing leucocytosis with a left shift, anaemia (Hct: 16%), hypoalbuminaemia, fever, and persistent ptyalism. Methadone and meloxicam were discontinued, and intravenous buprenorphine 0.02 mg/kg (Bupaq P 0.3 mg/mL, Streuli Pharma AG, Switzerland) was introduced for pain management. Large pockets of corpuscular fluid in the inguinal adipose tissue were observed via ultrasonography. Due to an uncertain prognosis and financial constraints, humane euthanasia was performed.

## 3. Discussion

This case describes advanced analgesic interventions for severe pain and highlights dosage recommendations, monitoring, and management of a CEI via an epidural catheter. In the medical field, extradural catheter placement is commonly performed in both adult and paediatric patients, with reported analgesic efficacy as high as 92.2% [4,6,13]. While veterinarians have access to similar techniques and resources, the placement of extradural catheters in animals, particularly cats, is uncommon. A review by Hansen et al. [7], examining 182 cases of extradural catheter analgesia in dogs and cats, found that only 12.1% involved cats, illustrating the relative rarity of this procedure in the species. This discrepancy may stem from technical challenges and anatomical differences involved in extradural catheter placement in cats compared to dogs. For instance, the dural sac extends more caudally in cats, reaching S1, compared with L6-7 in dogs [3]. For this reason, inadvertent dural puncture and intrathecal catheter placement are reportedly much higher in cats (27%) [7]. Additionally, the smaller size and delicate anatomy of cats further complicates catheter placement, likely limiting its use in veterinary practice.

The decision to perform extradural catheterisation at the L7-S1 intervertebral space was based on the need for adequate coverage of the caudal lumbar and sacral regions [14]. A premeasured distance to the L5 intervertebral space was used to guide catheter advancement. While sacrococcygeal extradural puncture is considered a safe alternative in cats [15], the authors considered maintaining long-term catheter sterility at this site likely more challenging. Subcutaneous tunnelling of the catheter could have been performed to reduce the risk of infection [16,17].

Correct catheter placement is crucial for effective analgesia and can be verified using imaging modalities such as radiography, fluoroscopy, or ultrasonography [3]. Other implications of inappropriate placement include inadvertent dural puncture, catheter coiling, and mechanical or chemical injury of the spinal cord [7]. In this case, contrast radiography was used to verify the catheter’s position. The spread of contrast from L3/4 to L7/S1 was observed, suggesting appropriate distribution of the local anaesthetic, although the catheter tip was not directly visualised. A larger volume of undiluted contrast could have enhanced visualisation, but in the authors’ experience, undiluted viscous contrast risks blockage of narrow lumened catheters. On the second day, catheter placement was verified using the injection of a fast-acting local anaesthetic, lidocaine, testing spinal reflexes and sensitivity pre- and post-injection. Aside from correct catheter positioning, the risk of kinking or breakage should be carefully monitored and minimized [7]. At the time of catheter removal, the insertion site appeared unremarkable, and the catheter was intact without any signs of mechanical failure.

Additionally, care must be taken as the initiation of a CEI and its attachment to an extension set can lead to dislodgement and compromise positioning during patient movement or manipulation [18]. To minimise this risk, the catheter extension set was secured to the top of the patient’s enclosure, allowing sufficient slack to allow movement. Again, subcutaneous tunnelling could have also further reduced this risk [16,17].

Initially, analgesia was achieved with an extradural bolus of 0.5% ropivacaine (0.13 mL/kg or 0.6 mg/kg), but the effects were short-lived, necessitating frequent repeat administrations. Transitioning to a CEI resulted in consistently low pain scores, avoiding the peaks and troughs associated with intermittent bolus dosing. Repeated boluses given more frequently than ropivacaine’s elimination half-life can elevate plasma concentrations of unbound drug, increasing the risk of toxicity [19]. In paediatric patients, unbound ropivacaine levels reach a steady state after 24 h of a CEI [4]. While this has not been confirmed in cats, achieving a steady plasma concentration may be expected with appropriate dosing. Based on these considerations and recommended ropivacaine doses in cats [1], a 0.5% ropivacaine CEI was initiated at 0.18 mg/kg/h or 0.036 mL/kg/h, aligning with an anecdotal recommendation of 0.02–0.05 mL/kg/h [3]. However, this contrasts with Hansen et al. [7], who reported using 0.125% bupivacaine at lower infusion rates of 0.006–0.02 mL/kg/h or 0.008–0.03 mg/kg/h. It is unclear, however, whether these rates were applied in dogs or cats.

Excessive administration of local anaesthetics can lead to local anaesthetic systemic toxicity (LAST). LAST occurs when excessive peak plasma concentrations of the unbound drug are reached, with toxicity thresholds varying between local anaesthetics. While the incidence of LAST in cats is unknown, several cases have been reported with the use of bupivacaine [10,20]. The risk applies to both bolus and CEI administration. While CEI administration may achieve more stable and lower plasma levels, potentially reducing toxicity, there is also a risk of drug accumulation over time. Toxic doses of ropivacaine in cats are extrapolated from canine studies, in which convulsions occurred at cumulative doses of 4.88 mg/kg and cardiovascular collapse at 42 mg/kg intravenously [1]. Therefore, the bolus dose of 0.6 mg/kg and the CEI rate of 0.18 mg/kg/h used in this case were well within the maximal recommended limits. Nevertheless, it is generally accepted that cats are more susceptible to local anaesthetic toxicity than dogs [8,9,10].

One day after extradural catheter removal, the cat’s condition worsened, prompting consideration of differential diagnoses such as LAST, sepsis from the surgical site or extradural catheter, or surgical dehiscence. Signs of LAST in cats include hypersalivation, mydriasis, reduced mental status, absent pupillary light reflex, tonic–clonic seizures, and cardiovascular compromise [8,10,20]. Although the cat’s general condition deteriorated, no specific neurological deficits or parameters associated with cardiovascular compromise were observed. Persistent hypersalivation was the most prominent clinical sign, perceived to be related to nausea. Although nausea, vomiting, and headaches have been reported in humans receiving local anaesthetics, these adverse effects are generally rare [21]. Similarly, in veterinary patients, LAST is rarely associated with hypersalivation and nausea [18]. Moreover, hypersalivation persisted for more than 24 h after discontinuing ropivacaine, making LAST a less likely cause. Nausea and hypersalivation are known adverse effects of opioid administration, which may better explain the clinical signs observed [1]. However, it remains difficult to determine whether these signs were primarily caused by the analgesic agents or another underlying issue. Some neurologic signs typically associated with LAST, such as convulsions and reduced mental status, were not observed in this cat. However, a complete neurologic examination, including pupillary light reflex assessment, was not performed during hospitalization. Mydriasis is frequently observed in cats in our intensive care unit, but is not specifically documented, as it can have multiple causes (e.g., opioid administration, stress). Implementing targeted neurologic assessments in cats receiving continuous local anaesthetic infusions may be beneficial in the detection of LAST.

The development of leucocytosis, neutrophilia with a left shift, and fever suggested sepsis as the more likely cause of deterioration. Sepsis may have arisen due to dehiscence of the surgical site, but may also have been related to the extradural catheter. Although highly speculative, opioid administration could also have contributed due to their immunomodulatory properties [22]. Although there are limited data, extradural catheter infections in veterinary medicine are considered rare and do not necessarily cause systemic infection [7,20,23]. While catheter tip cultures were not performed in this case, even positive cultures do not necessarily indicate systemic bacteraemia [24]. Once sepsis was suspected and cardiovascular compromise deemed possible, NSAID administration was discontinued to reduce the risk of renal injury. Overall, the clinical picture in conjunction with ultrasonographic findings suggested that sepsis, resulting from wound dehiscence, was the most likely cause of the cat’s decline.

## 4. Conclusions

In this case, a CEI of 0.5% ropivacaine at 0.18 mg/kg/h provided effective analgesia, as evidenced by reductions in CMPS-Feline and FGS scores. This approach was particularly beneficial in managing severe caudal abdominal pain, offering sustained analgesia with minimal fluctuation. However, this technique is technically demanding, requiring accurate catheter placement, strict asepsis, and continuous care in the intensive care setting. Hypersalivation and general deterioration, occurring after catheter removal, could have been related to LAST, but were more likely multifactorial, involving disease progression, opioid use, and sepsis. This case highlights the potential of CEI for sustained analgesia in cats, a species with limited available data on CEI applications. Further research into the pharmacokinetics and safety of local anaesthetics administered via boluses or CEI in both dogs and cats is warranted.

## Data Availability

The original contributions presented in this case are included in this article. Further inquiries can be directed to the corresponding author.

## Abbreviations

The following abbreviations are used in this manuscript:

CEI	Continuous Extradural Infusion
CMPS-Feline	Glasgow Composite Measure Pain Scale-Feline
FGS	Feline Grimace Scale
LAST	Local Anaesthetic Systemic Toxicity
